# Supplementary material for: Smad2/3 Activation Regulates Smad1/5/8 Signaling via a Negative Feedback Loop to Inhibit 3T3-L1 Adipogenesis
Source: Int J Mol Sci. 2021 Aug 6;22(16):8472. doi: 10.3390/ijms22168472 (PMC8395197; doi:10.3390/ijms22168472)
Supplement: Supplementary file 1 [file ijms-22-08472-s001.zip › ijms-1273314-supplementary.pdf]

Figure S1: TGFβ family gene expression in differentiating 3T3-L1 cells

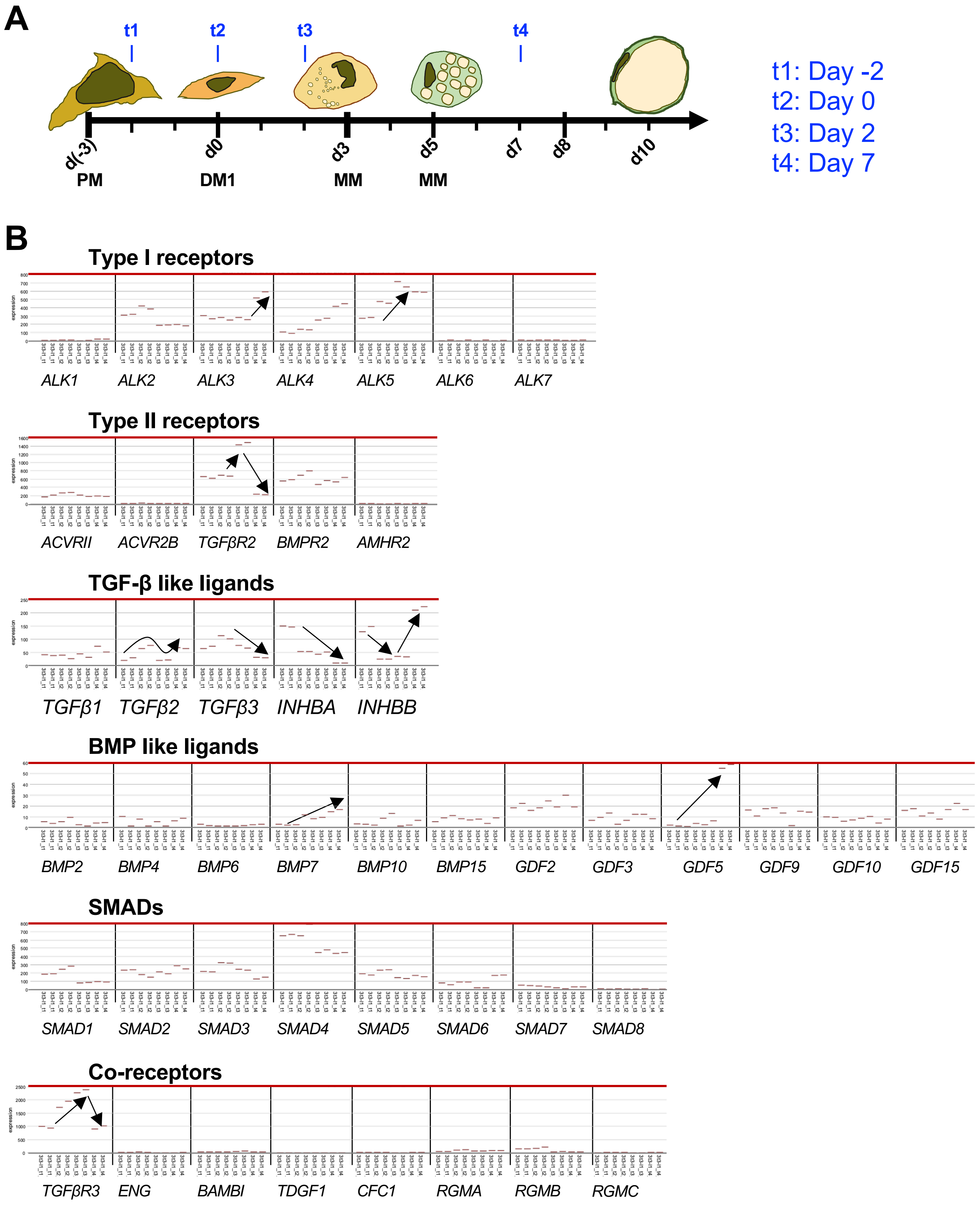

A) Gene expression analysis based on gse20696 data. Time points in study were taken on two days before differentiation medium (t1), during differentiation induction (t2), as well as two days (t3) and sevend days (t4) post differentiation. **(B)** The R2 web-based genomics analysis platform was used to analyze and display expression. Samples are separated by time point. Duplicate measurements are shown individually.

Figure S2: 3T3-L1 growth properties.

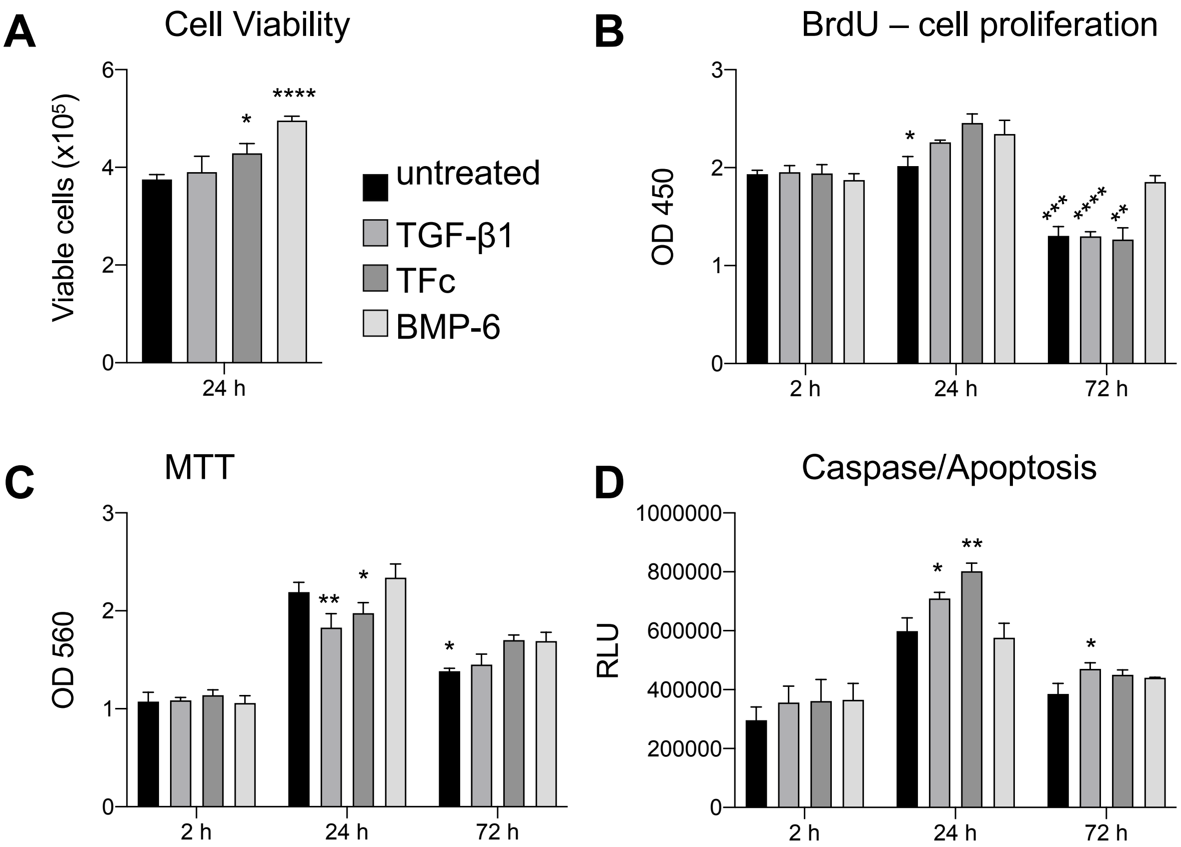

Experiments were carried out 2 hours, 24h hours or 72 hours post differentiation. Pre-adipocyte medium was changed at 0 hours into differentiation medium containing the control (black), TGF-β1 (medium grey), TGFβR2-Fc (TFc, dark grey) or BMP-6 (light grey). **(A)** Number of viable cells was determined at 24 hours post differentiation. **(B)** Apoptosis was measured using a fluorimetric Caspase-3 Immunoassay Kit. **(C)** cellular metabolic activity was measured using the MTT assay. **(D)** Proliferation was measured using a BrdU Cell Proliferation Assay. Statistical significance from four biological replicates was calculated by ordinary one-way ANOVA (Viability) and Bartlett's test or by Mixed-effects analysis and Tukey's multiple comparisons test (others) (* p<0.05; **p<0.01; ***p<0.001, ****p<0.0001). Reported statistical significance in A and B is relative to untreated controls (black). Reported statistical significance in C and D is relative to BMP-6 treated samples (light grey).

Figure S3: SMAD phosphorylation sites

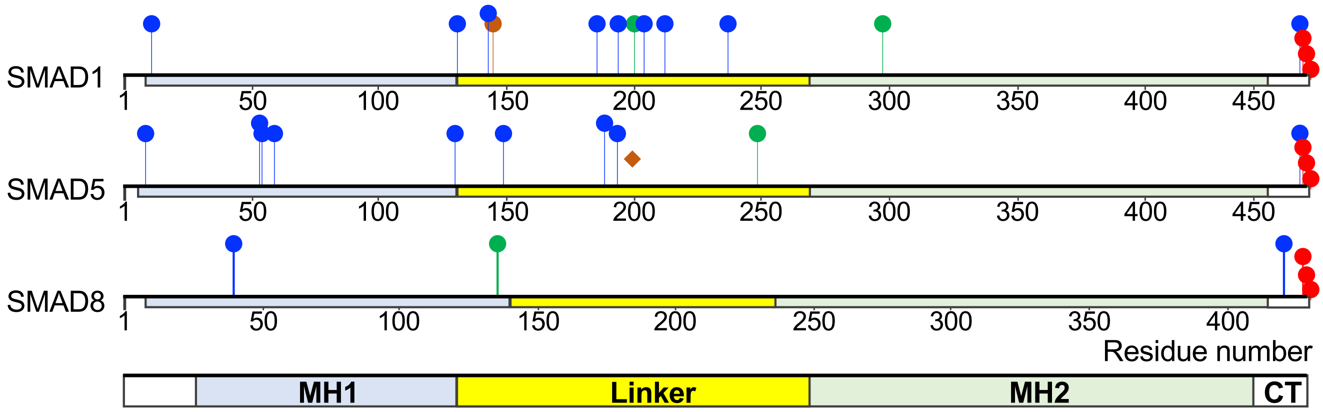

- MH stands for MAD homology and denotes a SMAD domain.
- Circles correspond to experimentally determined phosphorylation sites.
- Diamonds correspond to predicted sites (NetPhos 3.1 Server).
- Blue represents phosphorylated Serine residues.
- Green represents phosphorylated Threonine residues.
- Brown represents phosphorylated Tyrosine residues.
- Red represents C-terminal Serine residues phosphorylated in canonical TGFβ signal transduction.

Figure S4: Analysis of non-canonical pathway activation. Although several kinases mayhyper-phosphorylate SMADs at their interdomain linker region and ERK phosphorylation was increased when cell were treated with adipogenesis inhibitors, further analysis indicates that ERK kinase may not directly give rise to the p-SMAD1/5^{Hi} species in this context.

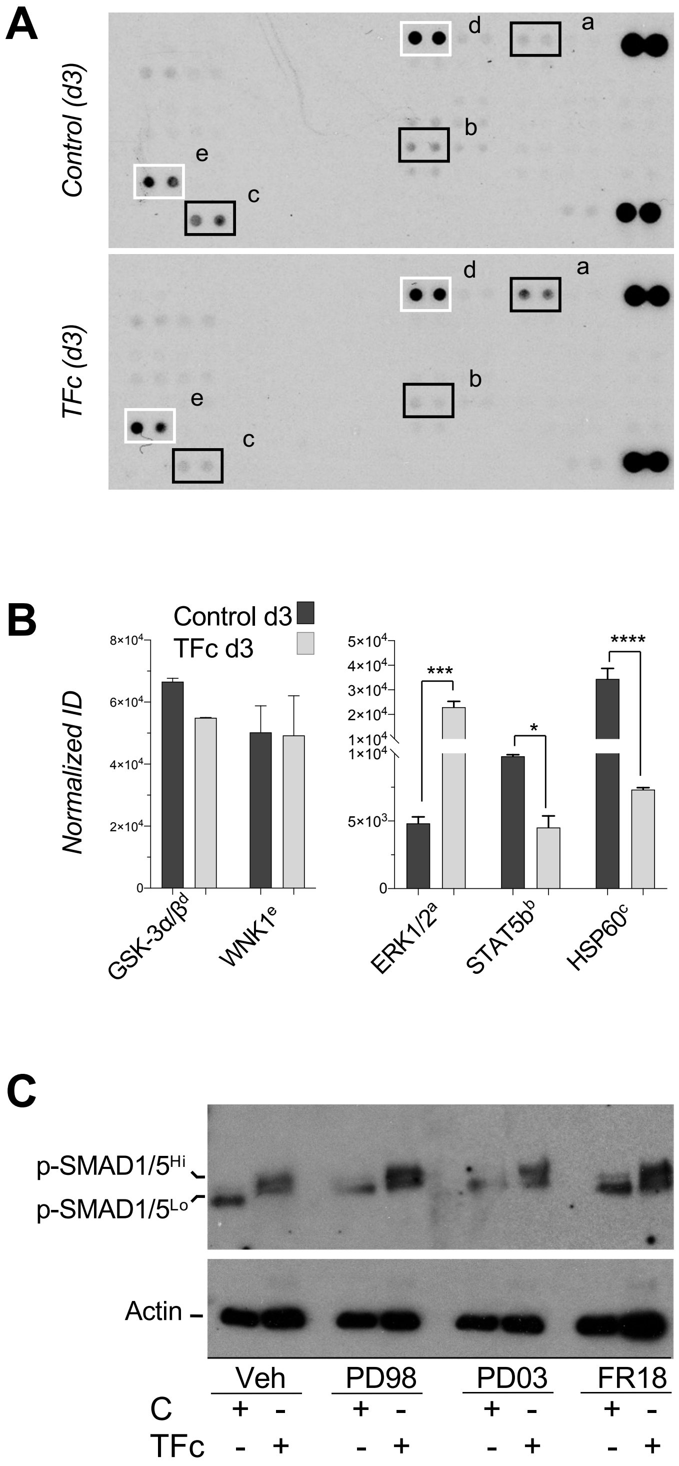

A) Analysis of kinase phosphorylation (Phospho-Kinase Array Kit, ARY003B) in Vehicle Control and TGFβRII-Fc treated samples collected at day 3 of differentiation. Black boxes represent proteins that are differentially phosphoryl-ated between Control (C) and TGFβRII-Fc (TFc) treated samples. White boxes represent proteins that are phosphoryl-ated in all 3T3-L1 samples. Box (a) corresponds to ERK1/2, box (b) to STAT5b, box (c) to HSP60, box (d) to GSK-3α/β and box (e) to WNK1. **(B)** Quantitative analysis of Kinase Array blots. Spot integrated densities were determined with ImageJ. Intensities were normalized against capture control antibody spots. Statistical significance from two biological replicates was determined by one-way ANOVA and Fisher's LSD tests (* $p<0.05$; ** $p<0.01$; *** $p<0.001$) using GraphPad. **(C)** Effect of ERK inhibitors on SMAD1/5/8 hyper-phosphorylation. Control (C) and TGFβRII-Fc (TFc) treated samples 3T3-L1 cells were simultaneously treated with vehicle (Veh, DMSO) or with the ERK inhibitors PD98059 (PD98, 1 μM), PD0325901 (PD03, 1 μM), and FR180204 (FR18, 10 μM). Equal amounts (10 μg) of protein from 3T3-L1 cells collected at day 3 of differentiation were analyzed by Western blot using a specific p-SMAD1/5/8 antibody. Actin loading controls are also shown (bottom panel). ERK inhibitors do not prevent formation of the p-SMAD1/5^{Hi} form.

Figure S5: Dual SB43 and LDN treatment on 3T3-L1 differentiation.

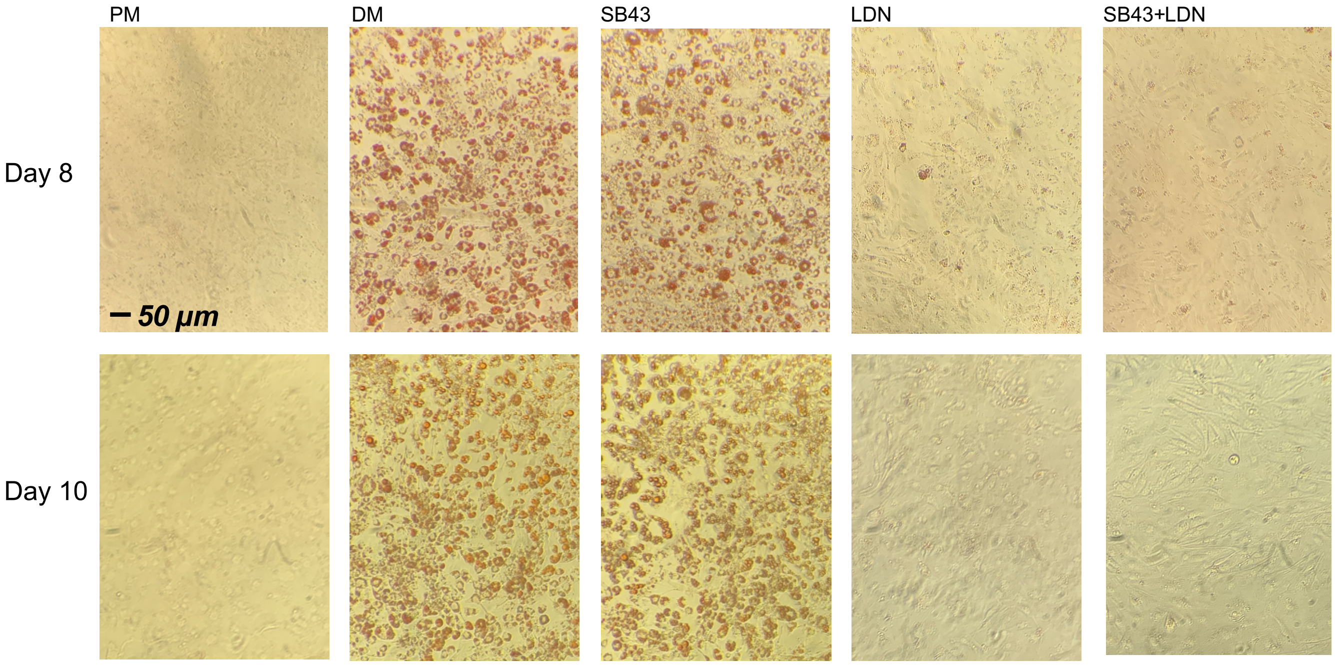

Oil-red-O images were taken at days 8 and 10 post differentiation. Undifferentiated cells (PM, pre-adipocyte medium), Differentiated Cells (DM, differentiation medium), as well as SB43, LDN and SB43+LDN treated cells in DM are shown.

Table S1: HUGO Gene nomeclature of TGFβ molecules

- ALK1: ACVRL1: activin A receptor like type 1
- ALK2: ACVR1: activin A receptor type 1
- ALK3: BMPR1A: bone morphogenetic protein receptor type 1A
- ALK4: ACVR1B: activin A receptor type 1B
- ALK5: TGFBR1: transforming growth factor beta receptor 1
- ALK6: BMPR1B: bone morphogenetic protein receptor type 1B
- ALK7: ACVR1C: activin A receptor type 1C

- ActRIIA: ACVR2A: activin A receptor type 2A
- ActRIIB: ACVR2B: activin A receptor type 2B
- BMPRII: BMPR2: bone morphogenetic protein receptor type 2
- TGFβRII: TGFBR2: transforming growth factor beta receptor 2
- AMHRII: AMHR2: anti-Mullerian hormone receptor type 2

- Cripto-1: TDGF1: teratocarcinoma-derived growth factor 1
- Cryptic: CFC1B: cripto, FRL-1, cryptic family 1B
- Cerberus: CER1: cerberus 1, DAN family BMP antagonist
- BAMBI: BAMBI: BMP and activin membrane bound inhibitor

Table S2: Surface plasmon resonance (SPR) based binding constants (M^{-1}) to Fc fusion ligand traps. Only the overall magnitude of the K_d value is shown.

K_d	BMPRII	ActRIIA	Bambi	ALK4	ALK3	ALK2	Cerberus	Cripto	mCryptic	TGFβRII	ActRIIB
Activin A	-9	-11	NB	-9	NB	NB	NB	NB	NB	NB	-11
Activin B	-10	-11	-4*	NB	NB	NB	-11	NB	-9	NB	-12
BMP-2	NB	NB	NB	NB	-10*	NB	-6	NB	NB	NB	NB
BMP-4	-9	-9	NB	NB	-10	NB	-9	-9	NB	NB	-9*
BMP-6	-8	-8	NB	NB	NB	-7*	-8	NB	NB	NB	-9
BMP-7	-8	-9	NB	NB	NB	-7*	-8	NB	NB	NB	-9
BMP-9	-9	-9*	NB	NB	NB	-7*	NB	NB	NB	NB	-10
BMP-10	-10	-10	NB	NB	NB	NB	NB	NB	NB	NB	-10
GDF-8	NB	-9	NB	NB	NB	NB	NB	NB	NB	NB	-9
GDF-11	-8	-10	NB	-8	NB	NB	-6	NB	NB	NB	-10
GDF-3	-9*	-10*	NB	NB	NB	NB	-7*	-7	NB	NB	-10*
TGF-β1	NB	NB	NB	NB	NB	NB	NB	NB	NB	-9	NB

- Starred values are the estimates from one ligand injection at 80 nM.
- All other values are derived from multiple injections and have been published.
- NB means ‘no binding’.
- 300-350 RU of Fc fusion proteins were captured.
- 800 RU of ALK2-Fc was captured.

Fc fusion proteins were captured on the sensor chip using a human IgG1-Fc binding antibody. Ligands were injected over Fc fusion receptors.

Table S3: Antibodies used in this work

Primary Antibody Name	Company	Catalog Number	Antibody type
P-SMAD2 S465/467 138D4	Cell Signaling	3108	mAb
P-SMAD1/5 S463/465 41D10	Cell Signaling	9516	mAb
SMAD1 XP® D59D7	Cell Signaling	12430	mAb
SMAD2 XP® D43B4	Cell Signaling	5339	mAb
SMAD3 C67H9	Cell Signaling	9523	mAb
SMAD5 D4G2	Cell Signaling	12534	mAb
SMAD5 EP619Y	Abcam	ab40771	mAb
SMAD8 (a.k.a. SMAD9)	Abcam	ab48011	polyclonal
P-p44/42 MAPK T202/Y204 XP® D13.14.4E	Cell Signaling	4370	mAb
P-p38 MAPK T180/Y182 XP® D3F9	Cell Signaling	4511	mAb
P-TAK1 T184/187 90C7	Cell Signaling	4508	mAb

Secondary Antibody Name	Company	Catalog Number
Anti-Rabbit	Cell Signaling	7074
Anti-mouse	R&D	HAF018
Anti-goat	R&D	HAF109
